# Emery‐Dreifuss muscular dystrophy type 4: A new* SYNE1* mutation associated with hypertrophic cardiomyopathy masked by a perinatal distress‐related spastic diplegia

**DOI:** 10.1002/ccr3.2140

**Published:** 2019-04-21

**Authors:** Mastroianno Sandra, Leone Maria Pia, Castellana Stefano, Palumbo Pietro, Paola Crociani, Russo Aldo, Di Stolfo Giuseppe, Carella Massimo

**Affiliations:** ^1^ Cardiovascular Department Fondazione IRCCS Casa Sollievo della Sofferenza San Giovanni Rotondo Italy; ^2^ Clinical Genetic Unit Fondazione IRCCS Casa Sollievo della Sofferenza San Giovanni Rotondo Italy; ^3^ Bioinformatics Unit Fondazione IRCCS Casa Sollievo della Sofferenza San Giovanni Rotondo Italy; ^4^ Neurology Unit Fondazione IRCCS Casa Sollievo della Sofferenza San Giovanni Rotondo Italy

**Keywords:** Emery‐Dreifuss muscular dystrophy type 4, hypertrophic cardiomyopathy, *SYNE1* mutation

## Abstract

Hypertrophic cardiomyopathy could be part of a more complex syndrome like Emery‐Dreifuss muscular dystrophy type 4. Genetic analysis allowed to identify a de novo heterozygous missense mutation in SYNE1 gene (chr6:152665253:G > C), supporting physician to reach a correct diagnosis in patient affected by cardiomyopathy associated with a difficult clinical scenario.

## 1 BACKGROUND

Spastic diplegia is a very common form of cerebral palsy all over the world. Many neurological disorders simulate spastic diplegia, and this can lead to misdiagnosis and inappropriate treatment. In our case, a young patient was treated for eighteen years as suffering from spastic diplegia due to perinatal distress until hypertrophic cardiomyopathy was discovered. Using a next‐generation sequencing (NGS) approach to interrogate the genome of the patient, we identify a new mutation in *SYNE1* gene (chr6:152665253:G > C). *SYNE1* encodes a member of the spectrin family of structural proteins that link the plasma membrane to the actin cytoskeleton. Mutations in this gene have been associated with musculoskeletal and cardiac diseases as the autosomal dominant Emery‐Dreifuss muscular dystrophy 4 (EDMD) and EDMD‐like phenotype. EDMD4 is characterized by muscular weakness and atrophy, with early joint contractures and cardiac abnormalities.

## 2 CASE REPORT

An 18‐year‐old girl is admitted to cardiology unit for precordial pain and dyspnoea.

**Figure 1:**
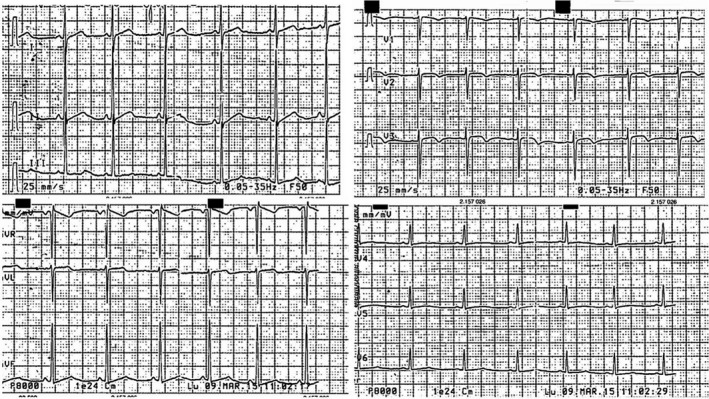
Electrocardiogram showing sinus rhythm with alterations of the ventricular repolarization as inverted T wave in V2‐V3, poor progression of the r wave in the precordial leads and high voltages in the peripheral leads

The patient appeared on a manual wheelchair for related neonatal suffering, with a scoliotic kyphosis, contraction of the elbows, and hypoactive left upper limb. She presented a hypotrophy of the bicuspid, tricuspid and gastrocnemius muscles and no signs of hemodynamic decompensation. She had negative family history of sudden death, cardiovascular, and neurological diseases. Her electrocardiogram presents a sinus rhythm with alterations of the ventricular repolarization as inverted T wave in V2‐V3, poor progression of the r wave in the precordial leads, maybe as a result of kyphosis, and high voltages in the peripheral leads (Figure 1). The echocardiogram performed at the time of admission shows asymmetric hypertrophic cardiomyopathy, particularly in the mid‐apical anterior wall (24 mm), in the absence of signs of left ventricular outflow obstruction, tricuspid aortic valve, and slight pericardial effusion, especially at anterior level, associated with epicardial fibrin deposits. Mitral, pulmonary, and tricuspid deficiency was mild.

The young patient was born prematurely at 31 weeks of gestation by cesarean section, and immediately after one hour, she developed respiratory distress and hypoglycemia and was admitted to neonatal intensive care and assisted by intermittent positive pressure ventilation. During the hospitalization, she was subjected to five blood transfusions for anemia, neonatal jaundice phototherapy and antibiotic and antifungal therapies for candida infection. The perinatal encephalic ultrasound allowed to identify small hemorrhage at the level of the caudate nucleus, bilateral peritrigonal hyperechogenicity, and an inhomogeneous area in the right thalamo‐striatal region of probable hypoxic‐ischemic nature. She was discharged from the neonatal hospital after 68 days with diagnose of prematurity and moderate asphyxiation.

At two years old, the child begins to present bilateral sensorineural hearing loss that suddenly gets worse after three years, for which she is performed to right cochlear implant and left retroauricular prosthesis.

The many brain MR performed before the implant showed a stationary neuroradiological image of alterations in bilateral periventricular and peritrigonal cerebral white matter by perinatal suffering.

Her clinical history and her lower limbs spasticity have led doctors to diagnose spastic diplegia and to start physiotherapy and orthosis treatments associated with focal therapy with botulinum toxin to reduce hypertonicity. During the years, physicians have witnessed a progressive worsening of walking, from a double support to the use of walker associated with orthopedic and orthotic shoes, and disturbed by equinovarism of the feet and global internal rotation of the lower limbs. At ten years, Achilles tendon extension surgery was also necessary to improve the gait of the small patient.

During cardiology admission, her laboratory tests were all within normal limits, including the serum CK level (69 U/l, normal range 26‐192). The Holter electrocardiogram showed rare single supraventricular extrasystoles and some monomorphic ventricular extrasystoles, with superior axis and right bundle branch block morphology, as arising from the left ventricle. Spirometry found a moderate degree of restrictive ventilatory deficit.

Given the complex clinical conditions, a genetic counseling was also recommended for the patient. She and her parents were recruited for a genetic screening by next‐generation sequencing (NGS) approach.

## 3 METHODS

### 3.1 Libraries preparation and Next‐generation sequencing

Genomic DNA was extracted from the blood samples of the proband using Bio Robot EZ1 (Quiagen).

The library preparation was performed using the TrueSight One Sequencing Panel kit (Illumina) which provides comprehensive coverage of >4800 disease‐associated genes (http://www.illumina.com/products/trusight-one-sequencing-panel.ilmn). The kit contains all the reagents necessary for the amplification, amplicon enrichment, and indexing of the samples. The sequencing of the library was performed on NextSeq 500 platform (Illumina). For the quantification and validation of the genomic library, the Qubit® 2.0 Fluorometer system (Life Technologies) was used. Each procedure was realized following the manufacturer's instructions.

The presence of the variant was checked in the proband and her parents by Sanger sequencing. PCR products were sequenced using ABI Prism 3100 Genetic Analyzer (Thermo Fisher Scientific) and the BigDye Terminator v1.1 sequencing kit (Applied Biosystems).

### 3.2 Bioinformatics analyses

Raw sequences were checked for their quality and mapped against the hg19 reference genome by Bowtie2.^1^ Quality check was performed by FastQC tool;^2^ in particular, adapters were removed using the exact match provided by the FastQC Adapter Content report. Coverage analysis was performed by the TEQC v3.4 R Package,^3^ while variants were called by means of the HaplotypeCaller tool of GATK ver. 3.4^4^ and were annotated with ANNOVAR, using RefSeq gene and transcript annotations.^5^ Annotated data were filtered out to exclude low functional variants, as well as variants with reported frequency <0.01 in publicly available human variation resources: dbSNP ver. 150,^6^ ExAC ver. 0.3, and gnomAD.^7^ Missense variants passing the previous filters were further investigated using dedicated software to assess their pathogenicity, the dbNSFP 3.3.^8^ Sample‐specific variants were also screened for their presence in the other sequenced samples within the same run.

## 4 RESULTS

**Figure 2:**
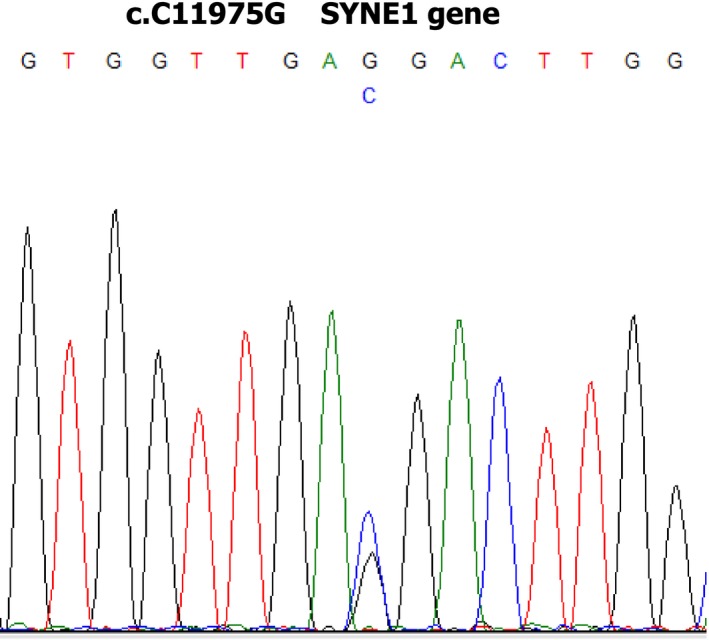
Electropherogram of the index patient showing the C.C11975G in the SYNE1 gene

Sequencing run yielded good quality throughput for the three investigated samples, with average base calling scores greater than 30 and average target coverage >170× (details in Table 1). Variant filtering was carried out giving priority to mutations that met the following criteria: detected as “novel” (undescribed in public databases) within the affected individual and not present in parents; annotated as “non‐synonymous/stoploss/stopgain/frameshift” mutation and not present in other sequenced samples. Among the 118 prioritized exonic variants, a SYNE1 mutation (chr6:152665253:G > C), encoding for a nonsynonymous substitution at amino acid position 3992 and 4063 (NM_033071:exon73:c.C11975G:p.P3992R; NM_182961:exon74:c.C12188G:p.P4063R) (Figure 2), resulted as the best candidate causative variant. It was called with very confidence, with phred‐like quality score higher than 1000 (ie, a score of 30 indicates a probability of being an artifact equals to 1/1000), with 55 out of 124 reads carrying the alternate allele. This de novo heterozygous mutation was not found within parents, or in public databases.

**Table 1:** Sequencing run throughput for the three investigated samples

	Proband (F)	Father	Mother
Generated paired‐end reads (million)	19.19	17.01	19.04
Mean read quality (phred‐like score)	33.04	32.91	33.11
Fraction on target	69.4%	69.45%	68.08%
Mean target coverage	199.31	175.02	189.54
SD target coverage	134.81	116.28	126.09
% of sites >20× coverage	89.29	87.75	88.79

## 5 DISCUSSION

Targeted region capture sequencing performed on the young patient affected by spastic diplegia revealed a heterozygous missense mutation in *SYNE1.* *SYNE1* gene comprises 516 Mbp on chromosome 6q25, contains 146 exons, and encodes for Nesprin‐1 (spectrin repeat‐containing nuclear envelope protein (a), a protein which belongs to the nesprin family of nuclear and cytoskeletal proteins.^9^ The mutation reported is localized into a central domain composed of multiple spectrin repeats (SR), which supports interactions with other proteins such as emerin, with the Emerin Binding Domain (EBD), or lamins, with the Lamin Binding Domain (LBD).^10^ This network of proteins physically interplays to guarantee nuclear envelop integrity and shapes the bridge between cytoplasmic cytoskeletons networks and the genome.^11^

Nesprin‐1 is ubiquitously expressed in multiple tissues, but it is particularly abundant in cerebellum and the striated muscles.^12^ Mutation in *SYNE1* could cause neurodegenerative disease such as autosomal recessive 8 spinocerebellar ataxia (OMIM#610743) and autosomal recessive myogenic arthrogryposis.^13^

*SYNE1* is also involved in pathogenesis of Emery‐Dreifuss muscular dystrophy (EDMD4,OMIM#612998) and EDMD‐like phenotype, which presents no significant cardiac abnormalities.^14^, ^15^

EDMD is a form of muscular dystrophy characterized by early‐onset joint contractures (elbows, ankles, and cervical spine), slowly progressive muscle weakness, and cardiac abnormalities characterized by cardiac conduction defects and dilated cardiomyopathy that occurs in late stage of disease and became the main cause of EDMD patient mortality.^16^

This may be attributed to myocardial cell replacement by fibrous and adipose tissue, starting in the atria and often involving the atrioventricular node with atrial arrhythmias, heart block, and progressive ventricular dilatation with systolic dysfunction.^17^

Mutations in *SYNE1* are associated with different cardiac phenotypes as dilated cardiomyopathy with conduction system defects,^18^, ^19^ but also with slight left ventricular basal and septal hypertrophy with mild diastolic dysfunction.^20^

The genetic screening of the patient by target resequencing approach (TRS) showed the presence of a de novo mutation in *SYNE1* gene. The mutation described here is the genetic cause the phenotype observed in our patient, which matched with the mayor EDMD clinical synopsis. In fact, she presented a constant worsening of symptoms due to the inability to walk, deformities of the spine as scoliosis, but he presented a nonobstructive hypertrophic cardiomyopathy form. For many years, the diagnosis of spastic diplegia masked EDMD and exposed the patient to an underestimation of arrhythmic risk and sudden cardiac death. Although only SYNE4 is described as strictly associated to deafness, we cannot exclude a possible implication of SYNE1 in progressive bilateral hearing loss since the first years of life, poorly explained by the ischemic cerebral damage alone.^21^

## 6 CONCLUSIONS

We reported a patient affected by Emery‐Dreifuss muscular dystrophy type 4, associated with hypertrophic cardiomyopathy and carrier of a novel de novo mutation in the *SYNE1* gene. Currently, only few cases of mutations in SYNE1 are reported to be responsible of EDMD. Comparing the current case with those previously described, we found that the patient's clinical manifestations, despite the signs of neonatal distress, can also be attributed to EDMD4.

In conclusion, we would like to focus how some infantile cerebral paralysis manifestations, such as spastic diplegia, can mask genetically determined pathologies at risk for life‐threatening arrhythmic complications. At same time, we underline the role of genetic analysis allowing the correct recognition of this demanding diagnosis, bringing together neurological and cardiological spectrum of this complex clinical scenario, leading to proper clinical management.

## CONFLICT OF INTEREST

None Declared.

## AUTHOR CONTRIBUTION

MS: has contributed to acquisition of clinical patient data, drafted and revised the manuscript, and gave the final approval for publication. LMP and CM: has contributed to acquisition of genetic patient data, drafted and revised the manuscript, and gave the final approval for publication. CS: has contributed to bioinformatic analysis of genetic data, drafted the manuscript, and gave the final approval for publication. PP: has contributed to acquisition of genetic patient data, drafted the manuscript, and gave the final approval for publication. CP: has contributed to patient neurological assessment, drafted the manuscript, and gave the final approval for publication. RA: has contributed to acquisition of clinical patient data, drafted the manuscript, and gave the final approval for publication. DSG: has contributed to acquisition of clinical patient data, drafted and revised the manuscript, and gave the final approval for publication. All authors agree to be accountable for all aspects of the work in ensuring that questions related to the accuracy or integrity of any part of the work are appropriately investigated and resolved.
